# Effects of Heavy Metals from Soil and Dust Source on DNA Damage of the *Leymus chinensis* Leaves in Coal-Mining Area in Northwest China

**DOI:** 10.1371/journal.pone.0166522

**Published:** 2016-12-09

**Authors:** Tianxin Li, Minjie Zhang, Zhongming Lu, Uwizeyimana Herman, Dzivaidzo Mumbengegwi, John Crittenden

**Affiliations:** 1 School of Civil and Environmental Engineering, University of Science and Technology Beijing, Beijing, China; 2 Brook Byers Institute for Sustainable Systems, Georgia Institute of Technology, Atlanta, Georgia, United States; 3 Research Center for Eco-Environmental Sciences, Chinese Academy of Sciences, Beijing, China; 4 School of Civil and Environmental Engineering, Georgia Institute of Technology, Atlanta, Georgia, United States; Jinling Institute of Technology, CHINA

## Abstract

Air and soil pollution from mining activities has been considered as a critical issue to the health of living organisms. However, few efforts have been made in distinguishing the main pathway of organism genetic damage by heavy metals related to mining activities. Therefore, we investigated the genetic damage of *Leymus chinensis* leaf cells, the air particulate matter (PM) contents, and concentrations of the main heavy metals (Pb, Cd, Cr, Hg) in soil and foliar dust samples collected from seven experiment points at the core mining area and one control point 20 kilometers away from the core mining area in Inner Mongolia in 2013. Comet assay was used to test the genetic damage of the *Leymus chinensis* leaf cells; the Tail DNA% and Tail Moment were used to characterize the genetic damage degree of the plant cells. The comet assay results showed that the cell genetic damage ratio was up to 77.0% in experiment points but was only 35.0% in control point. The control point also had the slight Tail DNA% and Tail Moment values than other experiment groups. The cell damage degree of the control group was 0.935 and experiment groups were 1.299–1.815. The geo-accumulation index and comperehensive pollution index(CPI) were used to characterize heavy metal pollution in foliar dust samples, and single factor pollution index and CPI were used to characterize the heavy metal pollution in soil samples. The CPI_foliar dust_ of control group was 0.36 and experiment groups were 1.45–2.57; the CPI_soil_ of control group was 0.04 and experiment groups were 0.07–0.12. The results of correlation analyze showed that Air Quality Index (AQI) -CPI_foliar dust_(r = 0.955**)>Damage degree-CPI_foliar dust_(r = 0.923**)>Damage degree-AQI(r = 0.908**)>Damage degree-CPI_soil_ (r = 0.824*). The present research proved that mining activity had a high level of positive correlation with organism genetic damage caused by heavy metals through comparing with the control point; soil and atmosphere were both the important action pathway for heavy metal induced genetic damage in mining area. Furthermore, heavy metal contents in foliar dust showed a higher positive correlation with genetic damage than when compared with soil. This means the heavy metal contents that *L*.*chinensis* absorbed through respiration from the atmosphere could make more serious genetic damage than when absorbed by root systems from soil in the mining area. This study can provide theoretical support for research on plant genetic damage mechanisms and exposure pathways induced by environmental pollution.

## 1.Introduction

With the spread of heavy “smog and haze”, the problem of environmental health impact is regaining worldwide concern. Especially in the northwest of China, with the expansion of the industrial scale, the large-scale development of mineral resources caused serious environmental pollution, in particular the haze problem caused by airborne particulate matter. Surface mining operations, collection, transportation and handling of coal are the major sources of such fugitive emission[1], and these emissions can contribute significantly to heavy metal contamination around mining areas[2]. Moreover, heavy metals from these emissions are easily absorbed by plants, animals and human beings exposed to these elements[3], and can cause genetic damage on some extent[4].

At present, the domestic and foreign research about heavy metal pollutions are mainly related to the health risk assessment of some small mammals, hydrobiose and plants. Guerrero-Castilla et al[5] determined the concentrations of several metals in liver tissue, as well as the status of different molecular markers (oxidative stress, metal exposure, DNA damage, xenobiotic metabolism) in wild mice near coal-mining operations which showed the mutation of genes in the liver of wild mice exposed to coal mining environments. Luqing et al[6] conducted comet assay and DNA alkaline unwinding assay on the tissues (gills, hepatopancreas, and hemocytes) of *Charybdis japonica* in order to illustrate genotoxicity of three heavy metal ions (Cu^2+^, Pb^2+^, and Cd^2+^) on marine crabs, and this research indicated that the DNA damage can be used as the potential biomarkers of heavy metal pollution in marine environments. Plants can absorb heavy metals from the atmosphere and soil by respiration and root uptake, making them a great reception and collector for heavy metals from external environment and have a variety of reactions. Thus plants have been widely used as the indicator of the heavy metal pollutants[7–9]. On the other hand, present researches on heavy metal pollution in mining areas are mainly focused on the soil heavy metal pollution. Mining activities and mineral processing can generate large volumes of metal-rich waste materials and be considered as the principal cause of heavy metal soil contamination[10–11]. Monterroso et al[12] identified metal-tolerant plants with potential application in phytoremediation strategies, evaluated the distribution and chemical fractionation of heavy metals in soils and their accumulation or exclusion by native plant species growing in an abandoned Pb/Zn mine in North Western Spain. Gjorgieva et al[4] investigated effects of heavy metal stress on DNA damages and total antioxidants level in *Urtica dioica* leaves and stems. The results suggested that heavy metal stress influenced antioxidant status and also induced DNA damages in *U*. *dioica* that may help to understand the effects of metals genotoxicity. However, with the atmospheric pollution issues getting more and more serious, the health risk of the organisms surrounding mining areas induced by these airborne heavy metal particles can not be ignored anymore. It needs to be researched that the major approach of organisms suffered from heavy metals around the mining area. Heavy metals may be accumulated by plants from soil or atmosphere[13]. The majority of studies carried out so far about heavy metal pollution on plants have been concerned with root uptake rather than foliar uptake[14–15]. Tianxin et al[16] observed that URE activity was stimulated by the increasing of traffic air pollutants and concluded that the degraded soil quality can negatively affect the target of developing plants, but there was no research evidence to determine the main pathway plant get damaged in that research. Little was known about the contribution of foliar uptake to genetic damage induced by the mining-related metals in plants. Therefore, studying the main pathway for plants genetic damaged in the mining area has very important significance for protecting the plants, animals, and human health living around the mining area.

*L*. *chinensis* is the main forage resource for Inner Mongolia grassland as an herbaceous perennial grass with rich nutrients. It is wide spread in this study’s coal mining area. *L*. *chinensis*, as a typical but not rare plant species, was chosen as a suitable bio-indicator since it is of low cost and suitable for easy sampling. We conducted an investigation on *L*.*chinensis* leaves collected from mining area to: (i) quantify the degree of DNA damage of the *L*.*chinensis* leaf cells, the air quality, the heavy metal (Pb, Cd, Cr, Hg) accumulation in soil and foliar dust; (ii) assess the heavy metal pollution in soil and foliar dust; (iii) analyze the correlation coefficient of the degree of damage in the leaf cells with air quality, and the heavy metal pollution in foliar dust and soil, and further identify the main genetic damage pathway of heavy metals in plants in mining area.

## 2.Materials and Methods

Ethics Statement: The sampling behavior of this study obtained the permission from the Erdos Environmental Protection Agency, and does not involve any private land or any rare plant species.

### 2.1 Study Area

**Fig 1:**
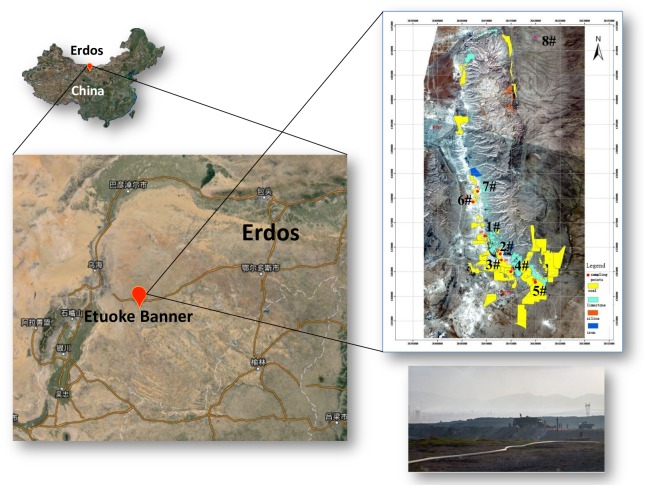
The study area location and sample site distribution.

The study area of this research is located in Etuoke Banner, west of Ordos in Inner Mongolia of China, between 106°41′ E ~ 108°54′ E and 38°18′ N ~ 40°11′ N. Coal mining is the pillar industry of Etuoke Banner, however, it caused heavy environmental pollution due to the long-term small-scale and dense layout mining activities, meanwhile with the outdated exploitation technology and misconduct of the pollutant emissions. The ecosystem of this mining area is very fragile. Vegetation is sparse, and difficult to restore once destroyed. This study selected seven sample sites and one control site (Fig 1). These sampling sites (1#~7#) located in core mining area, and the control site (8#) located 20 kilometers away from mining area. In addition, the control site (8#) located in the same environmental unit (with the same soil physicochemical properties) with 1#~7# sampling sites, but without any industry, coalmine and mining activities spread within 5 kilometers around, so, the 8# was chosen as the control point with no pollution. (The specific distributions are shown in S4 Table.)

### 2.2 Sampling and Treatment

#### 2.2.1 Plant, foliar dust and soil sampling

*L*.*chinensis* leaves, foliar dust and soil samples were collected from these 8 sampling sites (Fig 1) and their position fixed with the aid of a handheld GPS. At each sampling site, *L*.*chinensis* leaves (150~200g) with similar growth status were gathered by five-point sampling method. Then, the foliar dust (5~10g each sampling sites) was collected from the leaf samples with print papers and small brushes. These plant and foliar dust samples taken from each sampling site were homogenized before the analysis. Soil was dug to a depth ranging from 10 to 20cm. From each sample site, 10 sample points were selected randomly and the soil samples (0.5kg) were collected from each sample point, then subsequently mixed thoroughly and transferred to clean polypropylene bags.

In the laboratory, these *L*.*chinensis* leaves were cleaned of contaminants using a binocular microscope, and consecutive washings were applied with distilled water before the comet assay; foliar dust samples were stored in polyethylene bags at -20°C until they could be transported for analysis; and soil samples were air dried and sieved to <2mm, then ground in an agate mortar (grain size <50μm) to obtain consistency between the physical properties of the individual samples. Sufficient reproducibility of the digestion or extraction procedures was employed.

The sampling work was carried out over several sunny days, so the dust could deposit and accumulate on the foliar surfaces[17]. Plant, foliar dust and soil samples in this study were sampled once in August 2013.

#### 2.2.2 Atmospheric sampling

Based on the open-pit mine atmospheric investigation in former years[18], the concentration of H_2_S, SO_2_ and NO_2_ all achieved ‘Ambient Air Quality Standard’ (AAQS) secondary standard, with no violation. But the concentration of PM2.5(PM with aerodynamic diameter of less than 2.5 μm) and PM10(PM with aerodynamic diameter of less than 10 μm) exceeded standard badly. There were large amounts of heavy metal contents contained in these atmospheric particulates around coal mining area, which could induce genetic damage for plants and human beings. Thus, PM2.5 and PM10 were chosen to be the atmospheric monitoring factors for its rising public concern in recent years, and for the availability of environmental background information for analyzing the heavy metal content in foliar dust in the present research.

The concentration of PM2.5 and PM10 were detected from July to August in 2013 (with the exception of rain and snow days) during the sampling period. Samples were taken 2 meters from the ground within a circle of 2 meters around the sampling points and were detected from 8: 00hrs to 10: 00hrs. PM was measured over this period to assess the air quality.

### 2.3 Sample Detection and Pollution Quantification

#### 2.3.1 Comet assay

DNA damage of sample leaf cells was determined according to the comet assay described by Singh et al. (1988)[19] with slight modifications. After surface sterilization of the collected leaves, small pieces (1cm^2^) were cut. 10g of leaves were weighed and placed in a Petri dish kept on ice containing 40ml of grinding medium (20μmol/L sucrose, 10μmol/L MgCl_2_, 20μmol/L Tris-HCl buffer, pH7.8), and then the samples were ground into a homogeneous suspension using a mortar. The suspension was filtered using two filters (pore size are 61nm and 38nm). The final eluate was centrifuged for 5min at 200rpm (R = 10cm, G = 447.2g). The centrifuged solids were re-suspended in a buffer solution containing 1mmol/L MgCl_2_, 0.01M phosphate-buffered saline buffer (PBS), and S-buffer (1mol/L sorbitol, 25mmol/L phosphate buffer, pH6.5) and 1mmol/L phenyl methane sulfonyl fluoride (PMSF). After centrifugation for 10 min at 2000rpm (R = 10cm, G = 447.2g), sucrose solution was added into the centrifuged precipitate. The sucrose cushion contained 320mmol/L sucrose in 0.01MPBS and 1mmol/L CaCl_2_. Next, the centrifuged solids were re-suspended in S-buffer. It is recommended to perform the nuclear isolation in the dark to avoid white light induced damage.

The commonly used “sandwich” structure was chosen to perform the comet assay. Regular microscope slides were dipped into a solution of 1% normal melting point (NMP) agarose prepared with 1×PBS buffer at 65°C, and a cover slip was immediately placed on top. After the agarose layer solidified, the cover slip was removed. Then, 20μL of the cell suspension was mixed with 75μL of 0.8% LMP (low melting point) agarose and spread over the first layer. The cover slip was replaced on top, and the slide was incubated at 4°C for 10 min. Then, the cover slip was removed, 80μL of 1% LMP agarose was added as a third layer, and the cover slip was placed on top again until solidification. Then, the cover slip was removed, and the slide was immersed in freshly prepared ice-cold lysis solution (2.5mol/L NaCl; 100 mmol/L Na_2_EDTA; 10 mmol/L Tris; pH = 10; 1% sodium sarcosinate; 1%TritonX-100 and 10% DMSO were added immediately before use) for 1h at 4°C. The slide was drained and placed in a horizontal electrophoresis tank filled with freshly prepared alkaline buffer (300mmol/L NaOH; 1.0mmol/L Na_2_EDTA; pH>13) at 4°C for 30min. Electrophoresis was performed in this buffer for 20min by applying 20V and adjusting the current to 300mA. Finally, the slide was gently washed twice in a buffer solution (0.4mol/L Tris-HCl; pH = 7.5), fixed in methanol for 15 minutes, dried at room temperature, and stained with 0.005% ethidium bromide for 10 min. All procedures were carried out under low artificial light conditions to minimize UV-induced DNA damage. Slides were analyzed using a fluorescence microscope with an excitation filter (excitation 520nm, emission 590nm)[20]. A computerized image analysis system (CASP version 1.2.3b1, San Francisco, USA) was employed. From each sample, 200 cells were selected randomly for the assay, the number of damaged cells was counted, and statistical analysis of percentage of DNA in the tail (Tail DNA%) and Tail Moment was conducted as the DNA damage measures. Three slides were evaluated per treatment, and each treatment was repeated at least twice.

After unwinding and electrophoresis, the broken DNA will migrate out of the nucleus. Under the microscope, the images of damaged cells are observed as a bright comet head with a dispersive tail whereas healthy cells are observed as a round circle. Cell lysis solution can prevent damage to nucleic acids by nucleases, stabilize the structure of the nucleic acids, separate the nucleic acids and proteins, and then the proteins enter the gel and spread into the lysis solution, whereas the nucleic acids are retained. After dipping the samples in a strong alkaline electrophoresis solution (pH>13), healthy cells were round with a fluorescent, smooth surface (as shown in S4a Fig). If the cells were damaged, the DNA structure exhibits a fracture phenomenon, and the speed of fragment movement to the anode side is faster than the large segments during electrophoresis. Then, the DNA forms the comet phenomenon and has a clear tail (as shown in S4b Fig).

Viability was determined by the Trypan blue exclusion test. An aliquot of 50 μL cell suspension was mixed with 50 μL of 0.4% trypan blue solution. Then, 5 μL of the mixture was introduced in an improved Neubauer haemocytometer and cells (viable and non-viable cells) were determined microscopically. Cell viability always exceeded 95% in all samples.

#### 2.3.2 Atmospheric particulate analysis

Air quality index (AQI) is a quantitative description of air quality dimensionless index. In this research AQI was used to evaluate the dust pollution (PM2.5 and PM10) in study area, and using the following equations:

$${\text{IAQI}}_{\text{p}}=\frac{{\text{IAQI}}_{\text{Hi}}-{\text{IAQI}}_{\text{Lo}}}{{\text{BP}}_{\text{Hi}}-{\text{BP}}_{\text{Lo}}}\left({\text{C}}_{\text{p}}-{\text{BP}}_{\text{Lo}}\right)+{\text{IAQI}}_{\text{Lo}}$$

$$\text{AQI}=\text{max}\left\{{\text{IAQI}}_{1},\text{I}{\text{AQI}}_{2},{\text{IAQI}}_{3},\cdots {\text{IAQI}}_{\text{n}}\right\}$$

Where IAQI_P_ is the index for pollutant p; C_P_ is the mass concentration of pollutant p; BP_Hi_ is the largest observed value of C_P_; BP_Lo_ is the lowest observed value of C_P_; IAQI_Hi_ is the individual AQI value corresponding to BP_Hi_; and IAQI_Lo_ is the individual AQI value corresponding to BP_Lo_. The evaluation of AQI follow the Technical Regulation on Ambient Air Quality Index(HJ 633–2012). The specific classfications of the IAQI values are shown in S5 Table.

#### 2.3.3 Foliar dust heavy metal content detection and analysis

Foliar dust samples were freeze-dried (ALPHA 1–4/LD, Martin Christ Inc.) and mixed thoroughly using mortars and pestles. A small portion of each sample (0.5g) was passed through a 63mm sieve, weighed, and placed in a polytetrafluoroethylene vessel. The samples were hot digested with concentrated HClO_4_, HF, and HNO_3_ (Deng et al., 2010)[21]. The digested solutions were each diluted to 50 mL with 1:1 (v/v) HNO_3_ and stored in plastic bottles. Concentrations of Cd, Pb, and Cr were determined by a direct-reading inductively coupled plasma emission spectrometer analyzer (710-ES, Varian). Another 0.5g sample was passed through a 63mm sieve and digested for 2h with aqua regia (HNO_3_: HCl, 3:1) in a warm water bath (95°C). After cooling, the digestion solution was used for Hg measurement after being de-oxidated by a reducing agent (thiocarbamide and ascorbic acid). Concentrations of Hg were determined by an atomic fluorescence spectrophotometer (AFS-9230, Jitian Inc.). Blanks and sediment standard reference material (GSD-9, National Research Center for Certified Reference Materials, Beijing, China) were performed synchronously in each analytical batch. The recoveries were between 86 and 109%.

Evaluating pollution degree of the foliar dust samples. The geo-accumulation index (I_geo_) was introduced by Müller and has been widely employed in European trace metal studies[22–23]. It enables the assessment of environmental contamination by comparing differences between current and preindustrial concentrations. In this study, the I_geo_ for the foliar dust samples of examined mines was computed using the following equation:

$${\text{I}}_{\text{geo}}={\text{log}}_{2}\left(\frac{{\text{C}}_{\text{n}}}{1.5{\text{B}}_{\text{n}}}\right)$$

Where C_n_ is the measured concentration of every heavy metal found in the mine soil (mg/kg), and B_n_ is the geochemical background value of the heavy metals found in the soil (mg/kg). The constant 1.5 is used due to potential variations in the baseline data[24–25]. The geo-accumulation index consists of 7 grades: if I_geo_≤0, uncontaminated (class 0); if 0<I_geo_ ≤ 1, uncontaminated to moderately contaminated (class 1); if 1<I_geo_≤ 2, moderately contaminated (class 2); if 2<I_geo_≤3, moderately to heavily contaminated (class 3); if 3<I_geo_≤4, heavily contaminated (class 4); if 4<I_geo_ ≤5, heavily contaminated to extremely contaminated (class 5); if I_geo_ > 5, extremely contaminated (class 6).

#### 2.3.4 Soil heavy metal content detection and analysis

The freeze-dried soil samples for heavy metal analysis were finely pulverized and homogenized. Detection operation of the soil heavy metal content is the same as the detection on foliar dust samples.

In order to assess the soil contamination degrees, the comprehensive pollution index (CPI) was calculated. CPI can take into account all the functions of the pollutants in soil, and also highlights the influence of pollutants of high concentrations to the soil environmental quality, so we followed the same way to evaluate[26]. Based on the analysis of the single factor method pollution index (P_i_), we can obtain the CPI values.

P_i_ was calculated using the following formula:

$${\text{P}}_{\text{i}}=\frac{{\text{C}}_{\text{i}}}{{\text{S}}_{\text{i}}}$$

Where P_i_ represents the single factor pollution index of the pollutant i in the soil; C_i_ is the measured contamination value of the pollutant i; and S_i_ denotes the assessment standard of the pollutant i. Combined with the background of the study area, in this study, the P_i_s, calculated for four elements (Pb, Cr, Hg, and Cd) based on the third criterion of Environmental Quality Standard for Soils of China.

CPI was adopted to calculate the comprehensive pollution index of soil heavy metals and evaluate the pollution degree of soil in this study, following the Nemerow Pollution Index evaluation method defined in The Technical Specification for Soil Environment Monitoring (HJ/T 166–2004). The formula is expressed below:

$$\text{CPI}=\sqrt{\frac{{\left({\text{P}}_{\text{i}}\right)}_{\text{max}}^{2}+{\left({\text{P}}_{\text{i}}\right)}_{\text{ave}}^{2}}{2}}$$

Where CPI stands for the comprehensive pollution index of soil, (Pi)_ave_ represents the mean of various pollutant indices in the soil, and (Pi)_max_ is the maximum pollution index of single factor pollutant in the soil. If CPI≤0.7, the pollution degree of soil is cleaning and safety level; if 0.7<CPI≤1, the pollution degree of soil is slight cleaning but meeting warning limit level; if CPI>1, the content of soil heavy metal is beyond the third criterion of Environmental Quality Standard for Soils, which would influence the growth of farm crops, and affect human health; with if 1<CPI≤2 for slightly polluted level, 2<CPI≤3 for moderately polluted level, and CPI>3 for seriously polluted level.

### 2.4 Statistical Analysis

All values are expressed as mean±S.E. of six replicates in one representative experiment. The analyses were carried out using SPSS software (Version13.0). Data were compared with one-way ANOVA and Dunnett’s test. Statistical significance was defined as P<0.05 throughout.

## 3.Result and Discussion

### 3.1 Result of Comet Essay

#### 3.1.1 Tail DNA% analysis

**Fig 2:**
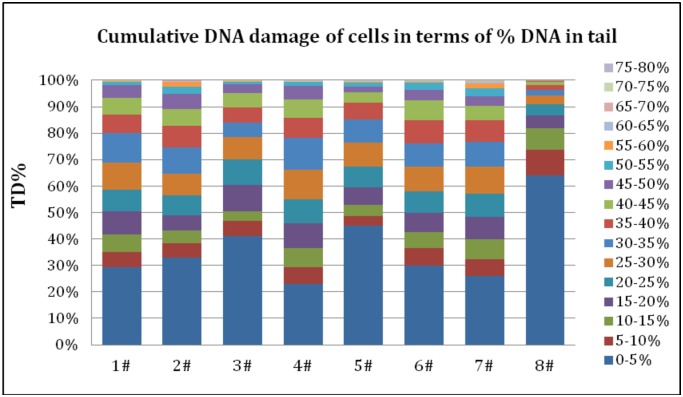
Cumulative DNA damage of cells in terms of Tail DNA%. *Not*:*1#~7#* *are sample points*; *8# is the control point*.

According to the comet images, the number of cells with a tail out of every 200 cells from each sample was counted. Based on the visual scoring of comet appearance, we identified different magnitudes of damage in the sampled population. According to the results, the maximum value for Tail DNA% (TD%) was 78.32 and the TD% was divided into 16 groups, 0–5%, 5%-10%, 10%-15% …… 70%-75%, 75%-80%. (Fig 2 and S1 Table).

The TD% reflects the proportion of DNA damage in the assayed cells. When TD% value is between 0%-5%, it could be regarded that there was almost no DNA damage in these cells. According to Fig 2, cell damaged ratio was 36.0% (TD% above 5) in the control group(8#), but was up to 55.0%-77.0% in other experiment groups, the 4# were most seriously damaged.

Comparing the proportion of cells in slightly damage (TD%<30%), the control sample was more than 85%. And there was no cell’s TD% above 45% in the control sample. Among these seven experiment points, the 4# was the most serious damaged group, the proportion of cells in low damage was less than 25%, and the proportion of cells above slightly damage (TD%>30) was almost 35%. As a result, these seven experiment samples showed a greater number of nuclei with medium damage and some nuclei with substantial damage (TD%>50%).

Compared to control samples (8#), the seven samples at the core mining area had much higher damage because of the difference in growing environment, and the biggest difference is whether the plant is growing around the coalmine plot. Therefore, the results indicated that living near the coal mine could cause *L*.*chinensis* DNA damage.

#### 3.1.2 Tail moment and DNA damage analysis

**Fig 3:**
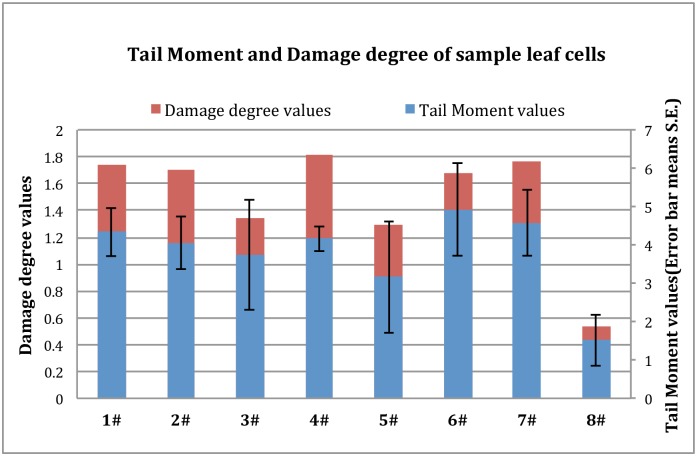
Tail moment and damage degree in the leaf sample cells.

The same cells from each group were chosen for tail moment analysis. As Tail Moment (TM) is an integrated value of the tail DNA density multiplied by the migration distance, different magnitudes of DNA damage are depicted in Fig 3, in which the abscissa corresponds to the sampling points and the ordinate corresponds to TM values.

The TM values represent the degree of damage in each cell. The seven testing sample groups exhibited higher TM values compared to the control sample(8#, p<0.01). Among the seven test samples, there was some variation, although the differences were not significant (p>0.05).

Considering the damage to each cell, this study calculated each group’s DNA damage degree. TM values were divided into five grades: Grade 0, when TM = 0; Grade 1, when TM ≤ 2; Grade 2, when TM ≤ 3; Grade 3, when TM ≤ 4; Grade 4, when TM > 4. Grade 0 represents the healthy cells.

The ratio of damaged cells in each grade was calculated as the damaged cell count divided by the total cell count. The damage degree of each sample was calculated using the formula below, and the results are shown in Fig 3.

$$\text{Damage degree}=\sum \frac{\left(\text{the grade }*\text{ cell number of each grade}\right)}{\text{number fo total testing cells}}$$

The results of the Damage degree in the leaf sample cells showed that the sample plant cells near the core mining area were more damaged than control samples (8#). The damage degree of the eight sample groups decreased in the order: 4#(1.815)>7#(1.765)>1#(1.742)>2#(1.701)>6#(1.678)>3#(1.349) >5#(1.299) >8#(0.935). The avarage damage degree of these seven test samples is 1.621, much more than the damage degree of 8#(0.935). Therefore, we can deem that mining activity could make a certain degree of influence on the genetic damage of plants around coalmine.

The results of the cell DNA damage degree further verified the mining activities can induce genetic damage to surrounding plants.

### 3.2 Result of Atmospheric Particulate Analysis

The PM results and AQI values are listed in Table 1. By constrasting the real measure of the PM density observation, we can find that the PM2.5 and PM10 concentration in the control site (8#) was significantly less than the sample sites (1#~7#). The AQI value of 8# was 60, meaning air quality was good in the control site; but the dust pollution was generally serious in other sample sites. The AQI values of 2#, 4#, 6# and 7# were 201~300, which is within the fifth class of air quality index, this means dust pollution was severe in these sample sites. 1# had the highest AQI value up to 326, dust pollution in this sample site was very serious. The results of the dust pollution analysis proved that coal mining activities can result in rather serious dust pollution in mining area, which has been proposed by many researchers[1][27].

**Table 1:** Dust pollution analysis in the sampling vicinity (unit: μg/m^3^). *Not*: *The assessment of AQI based on the PM2*.*5 and PM10*, *and the evaluation of AQI follow the Technical Regulation on Ambient Air Quality Index(HJ 633–2012)*.

Sample site	1#	2#	3#	4#	5#	6#	7#	8#
**PM2.5**	276±56	213±34	139±23	249±55	116±32	165±18	178±11	39±9
**PM10**	428±78	219±56	169±34	403±66	226±34	246±54	226±46	69±15
**AQI**	326	263	184	299	151	215	225	60

### 3.3 Foliar Dust Heavy Metal Content Assessment

**Fig 4:**
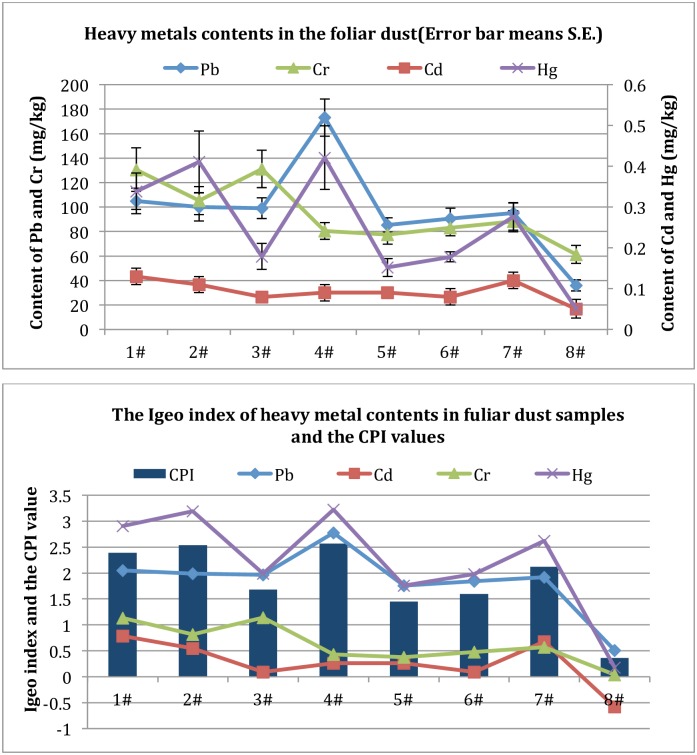
Spatial distribution of heavy metal levels in foliar dust samples. Not: The Igeo index was based on the soil background values of Inner Mongolia, according to the research data from the Inner Mongolia environmental monitoring center (2000).

The heavy metal contents and the I_geo_ values of the foliar dust samples are listed in Fig 4 (S2 Table). The distribution of I_geo_ values of the four heavy metals in foliar dust samples based on the soil background values of Inner Mongolia. The comprehensive pollution index (CPI) of the foliar dust for each sample points was presented in Fig 4.

The average contents of Pb, Cd, Cr, and Hg in foliar dust of the seven testing samples were much higher than those in the control group. The CPI_foliar dust_ values of 4#, 2#, 1# and 7# were much higher than other experiment groups, meaning the degree of damage caused by heavy metals in foliar dust were much more serious in 4#, 2#, 1# and 7#. In view of the I_geo_ values, Hg has the highest pollution degree; Pb is next; the lightest pollution element is Cr, which means plant leaves may be damaged by Pb and Hg particulate matters enriched in foliar dust through respiration at these sampling areas. Besides, through the correlation analysis, the concentrations of the Pb, Cd, and Hg in these foliar dust sample groups significantly correlate with each other (P<0.05).

### 3.4 Soil Heavy Metal Content Analysis

**Fig 5:**
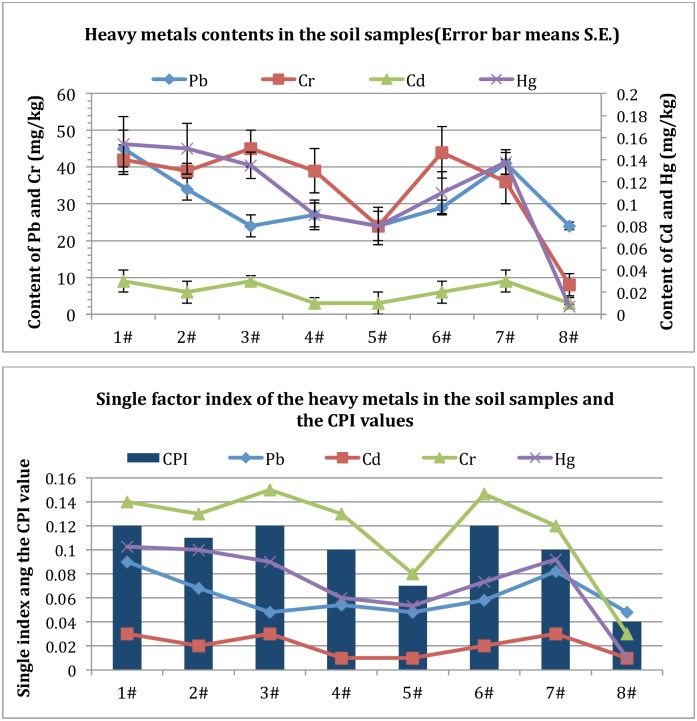
Spatial distribution of heavy metal levels in soil samples. Not: Using third grade standard of soil environmental quality standard as evaluation standard (GB15618-1995).

The total concentrations of heavy metals in soil samples from the coal mine are show in Fig 5. Combined with the background of the study area, this research chose the third grade standard of soil environmental quality standard (GB15618-1995) to evaluate the environment quality conditions. Evaluation results of single factor index for soil pollution by heavy metals and the evaluation result of the comprehensive pollution index (CPI) method are shown in Fig 5 (S3 Table).

The soil heavy metal pollution analyses showed that, the CPI_soil_ for these eight sample groups were all less than 0.15. It showed that according to the national third grade standard of soil environmental quality evaluation, soil in the study area was not polluted by these four kinds of heavy metals. Sample 8# had the lowest CPI_soil_ value(0.04), meaning the control group was in the lowest soil pollution degree compared with other experiment groups. The CPI_soil_ values of 3#, 6#, 1# and 2# were much higher than other experiment groups; meaning the damage degree of heavy metals in soil was much more serious in 3#, 6#, 1# and 2#. According to the single factor pollution index, different heavy metals had different distribution in each sample point, and the same heavy metal also had different distribution in different sample points. Through the correlation analysis on Pb, Cd, Cr, and Hg concentrations, the concentrations of the Pb, Cd, and Hg in these soil sample groups are also correlated significantly with each other (P<0.01).

### 3.5 Correlation Analyses

The correlation environment factors have among each other provided valuable information on their sources and behaviours[28]. Due to this reason, it is a method frequently employed in data evaluation. In the present study, the Pearson correlation coefficients and significance levels of the sample leaf cells damage degree, AQI, and the heavy metal contents obtained from the foliar dust and soil samples collected from these eight sampling sites, were calculated and are presented in Table 2.

**Table 2:** Pearson correlation coefficient results (r) of the AQI, CPI_foliar dust_, CPI_soil_ and leaf cells damage degree *Not*: **Correlation is significant at the 0.*01 level (P<0*.05; N = 8; two-tailed); * Correlation is significant at the 0.*05 level (P<0*.*05; N = 8; two-tailed)*.

Variable	Correlation coefficient			
	AQI	CPI_foliar dust_	CPI_soil_	Damage degree
AQI	1	0.955**	0.778*	0.908**
CPI_foliar dust_		1	0.740*	0.923**
CPI_soil_			1	0.824*
Damage degree				1

The positive correlations between the pairs of factors are Damage degree-AQI (r = 0.908), and Damage degree-CPI_soil_ (r = 0.824), it means the damage degree of sampling *L*.*chinensis* leaf cells has a higher correlation with the air quality compared with the soil pollution degree in the coal mining area. Considering the main air pollutants are heavy metal particles and coal fly dust in the mining area, and these particulates could settle on the blade surface of vegetation. Existing research[29] shows that the length of the *L*.*chinensis* leaf surface porosity between 37~42μm, and the width between 19~23μm, which means that the leaf surface stomatas of the *L*.*chinensis* could permit some nanoscale particles to access. Therefore, only nanoscale heavy metal particles may attach onto the surface of the leaf and will enter the leaf stomata, futher into the mesophyll tissue through respiration, and induce the genetic damage of plants.

Analyzing the positive correlations between the pairs of AQI-CPI_foliar dust_ (r = 0.955), and Damage degree-CPI_foliar dust_ (r = 0.923) has shown that the heavy metal contents in the foliar dust samples can be considered to be the main air pollutants in the study mining area. These heavy metals settled on foliar as dust also have a high correlation with the damage degree of sampling *L*.*chinensis* leaf cells. Which confirmed that lots of nanoscale heavy metal particles in the atmosphere can be absorbed by plant leaves and cause genetic damage to plants.

Comparing the positive correlations between the pairs of Damage degree-CPI_foliar dust_ (r = 0.923) and Damage degree-CPI_soil_ (r = 0.824), it can be said that heavy metals contained in the foliar dust have a higher level correlationship with leaf cells damage degree compared with heavy metals contained in soil. This may be because the heavy metal particles in the fly dust in mining areas accumulated in high concentration in a short time on the blade surface, and with the enrichment, lots of nanoscale particles could be absorbed by the leaf through micrometer size leaf stomatas during respiration, leading to enriched mesophyll tissue and induced genetic damage.

In general, these heavy metals contained both in foliar dust and in soil all exhibited a high positive correlation with the damage degree of sampling *L*.*chinensis* leaf cells. This showed that the soil and the atmosphere are both important pathways for plant genetic damage induced by heavy metals. Comparing these two pathways, heavy metals within the atmosphere have a more serious genetic toxicity than when found in soil.

## 4.Conclusion

Our results firstly focus on whether the atmosphere or the soil is the main pathway of plants genetic damage in mining areas. According to Comet Assay analysis, the damage ratio in the control group was 36.0%, in experiment (sample) groups up to 77.0%; and the damage degree in control group was 0.935, in experiment groups up to 1.815. The results proved that coal-mining activities dose relevant to plants genetic damage nearby coalmine in certain degree.

By analyzing the correlation coefficient of the genetic damage degree, the particulate matter contents, and the heavy metal pollution of the foliar dust and soil, it can indicate that the soil and atmosphere are both important pathways for plant genetic damage induced by heavy metals in mining areas. Comparing these two pathways, heavy metals contained in the atmosphere (Damage degree-CPI_foliar dust_(r = 0.923**)) had a more severe genetic toxicity than in soil (Damage degree-CPI_soil_ (r = 0.824*)). This may be because the heavy metal particles in the fly dust in mining areas accumulated. This shows that the genetic toxicity induced by heavy metals in plants through leaf uptake is much more serious than root uptake. Futhermore, due to the aggravating atmospheric pollution degree, the genetic damage induced by heavy metal contents on plant health through respiration exposure pathways would exceed root system absorbtion pathways, thereby elevating respiration to the principal action pathway to plant cell damage.

However, the direct relationship between DNA damage and these four metals (Pb, Cd, Cr, Hg) should be further investigated. Overall, the results can lay a foundation for further study of heavy metal pollution pathway related to mining activities, and provide a certain theoretical guidance for working direction on pollution control, plants and human health protection in and around mining areas.

## Supporting Information

S1 Fig Pictures of seven sample locations(1#~7#) in core mining area and one sample location (8#) away from the coal mining in Etuoke Banner. (PDF)

S2 Fig A total of 200 cells were scored for each sample from the captured images using the comet assay software project (CASP version 1.2.3b1). The number of damaged cells was counted, and statistical analysis of Tail DNA% and Tail Moment was conducted as the DNA damage measures.
(PDF)

S3 Fig Images of cell’s comet examined with a light microscope. If the cells were damaged, the DNA structure exhibits a fracture phenomenon, and the speed of fragment movement to the anode side is faster than the large segments during electrophoresis. Then, the DNA forms the comet phenomenon and has a clear tail.
(PDF)

S4 Fig Images of the healthy and damaged cell’s comet. After dipping the samples in a strong alkaline electrophoresis solution (pH>13), healthy cells were round with a fluorescent, smooth surface (as shown in S4a Fig). If the cells were damaged, the DNA structure exhibits a fracture phenomenon, and the speed of fragment movement to the anode side is faster than the large segments during electrophoresis. Then, the DNA forms the comet phenomenon and has a clear tail (as shown in S4b Fig).
(PDF)

S1 Table Cumulative DNA damage of cells in terms of Tail DNA%. (XLSX)

S2 Table Main heavy metals content in foliar dust samples (mg.kg^-1^dry weight) *Not*: *BC means the soil background values of Inner Mongolia*, *according to the research data from the Inner Mongolia environmental monitoring center (2000)*. (XLSX)

S3 Table Main heavy metals content in soil samples (mg.kg^-1^dry weight) *Not*: *Using third grade standard of soil environmental quality standard as evaluation standard (GB15618-1995)*. (XLSX)

S4 Table Description of sample site location. Not: 1#~7# are sample sites, located in the core mining area; 8# is the control site, away from the mining area.
(XLSX)

S5 Table The classfication of IAQI (based on HJ 633–2012) *Not*: *The evaluation of AQI follow the Technical Regulation on Ambient Air Quality Index(HJ 633–2012)*. (XLSX)
