# Supplementary figures and images for: Effects of Heavy Metals from Soil and Dust Source on DNA Damage of the Leymus chinensis Leaves in Coal-Mining Area in Northwest China
Source: PLoS One. 2016 Dec 9;11(12):e0166522. doi: 10.1371/journal.pone.0166522 (PMC5147816; doi:10.1371/journal.pone.0166522)

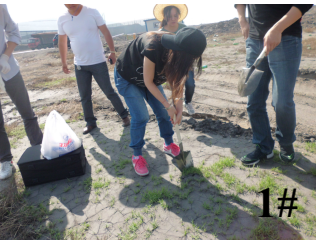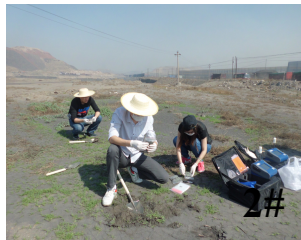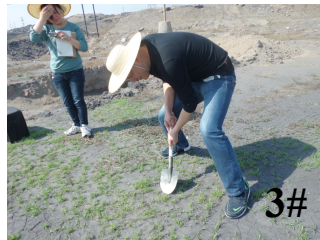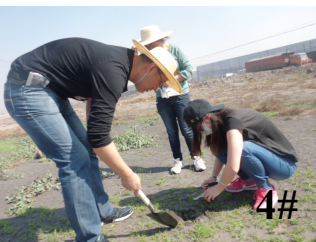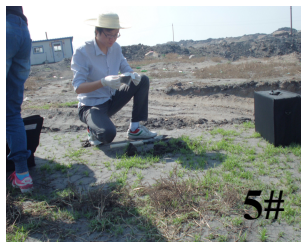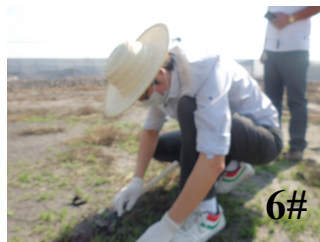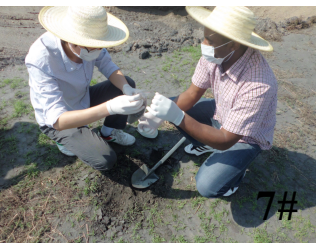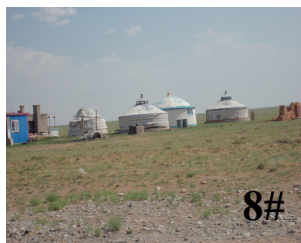

Supplement: S1 Fig — (PDF) [file pone.0166522.s001.pdf]

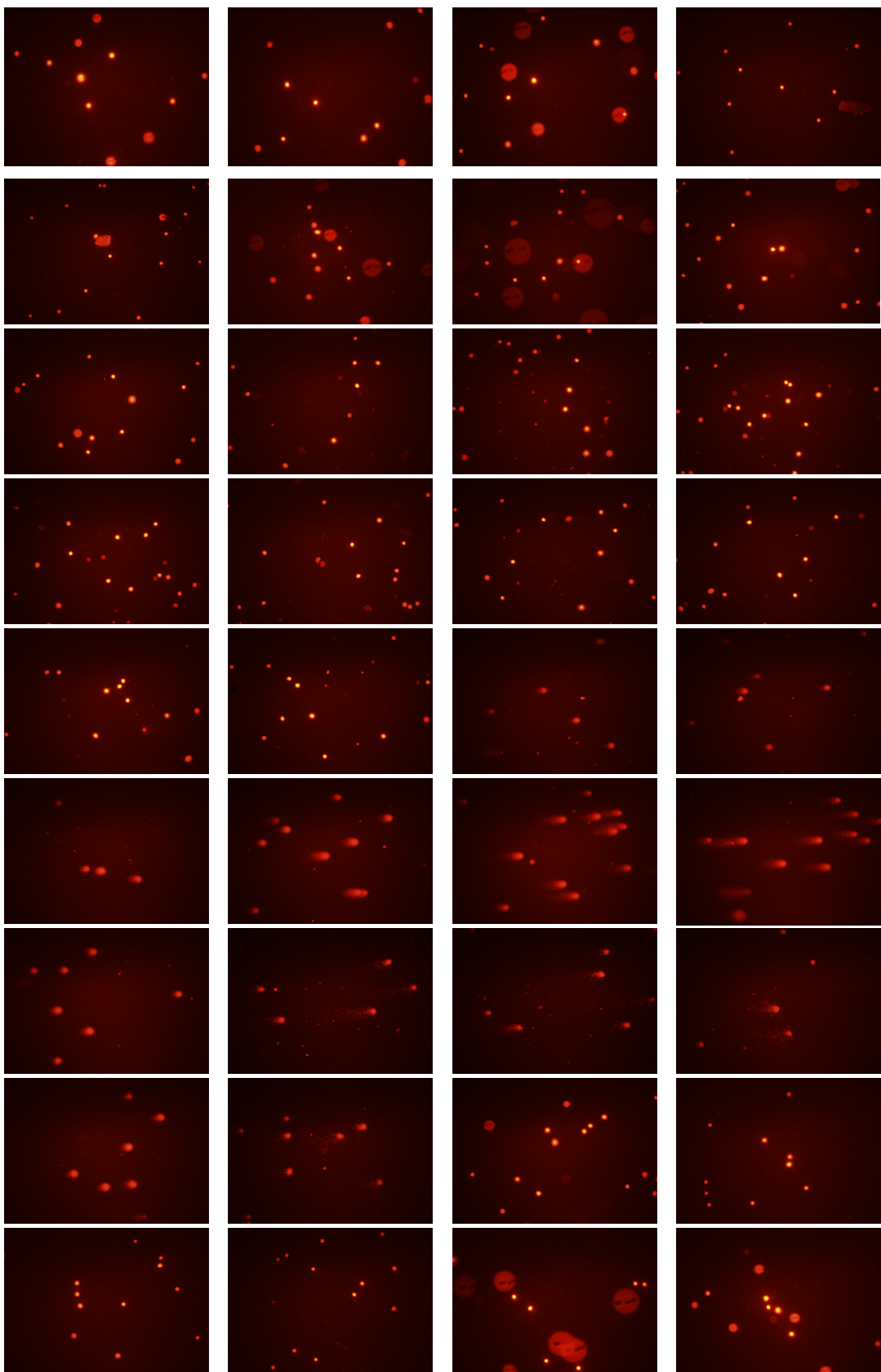

Supplement: S3 Fig — If the cells were damaged, the DNA structure exhibits a fracture phenomenon, and the speed of fragment movement to the anode side is faster than the large segments during electrophoresis. Then, the DNA forms the comet phenomenon and has a clear tail. (PDF) [file pone.0166522.s003.pdf]

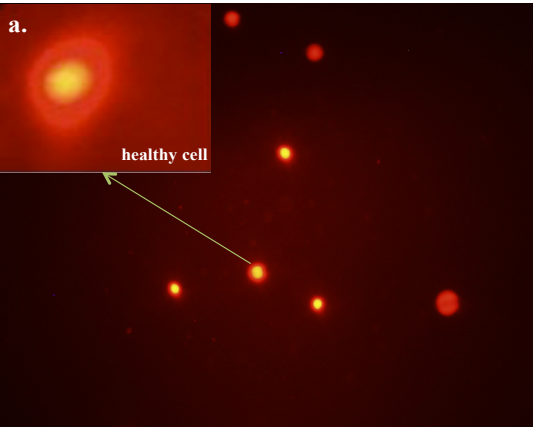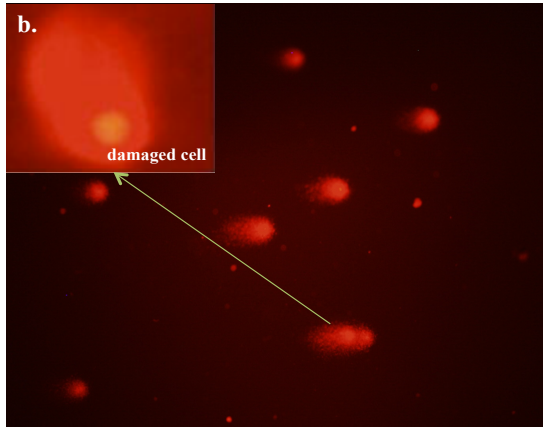

Supplement: S4 Fig — After dipping the samples in a strong alkaline electrophoresis solution (pH>13), healthy cells were round with a fluorescent, smooth surface (as shown in S4a Fig). If the cells were damaged, the DNA structure exhibits a fracture phenomenon, and the speed of fragment movement to the anode side is faster than the large segments during electrophoresis. Then, the DNA forms the comet phenomenon and has a clear tail (as shown in S4b Fig). (PDF) [file pone.0166522.s004.pdf]
