# Supplementary material for: Effects of Heavy Metals from Soil and Dust Source on DNA Damage of the Leymus chinensis Leaves in Coal-Mining Area in Northwest China
Source: PLoS One. 2016 Dec 9;11(12):e0166522. doi: 10.1371/journal.pone.0166522 (PMC5147816; doi:10.1371/journal.pone.0166522)
Supplement: S2 Fig — The number of damaged cells was counted, and statistical analysis of Tail DNA% and Tail Moment was conducted as the DNA damage measures. (PDF) [file pone.0166522.s002.pdf]

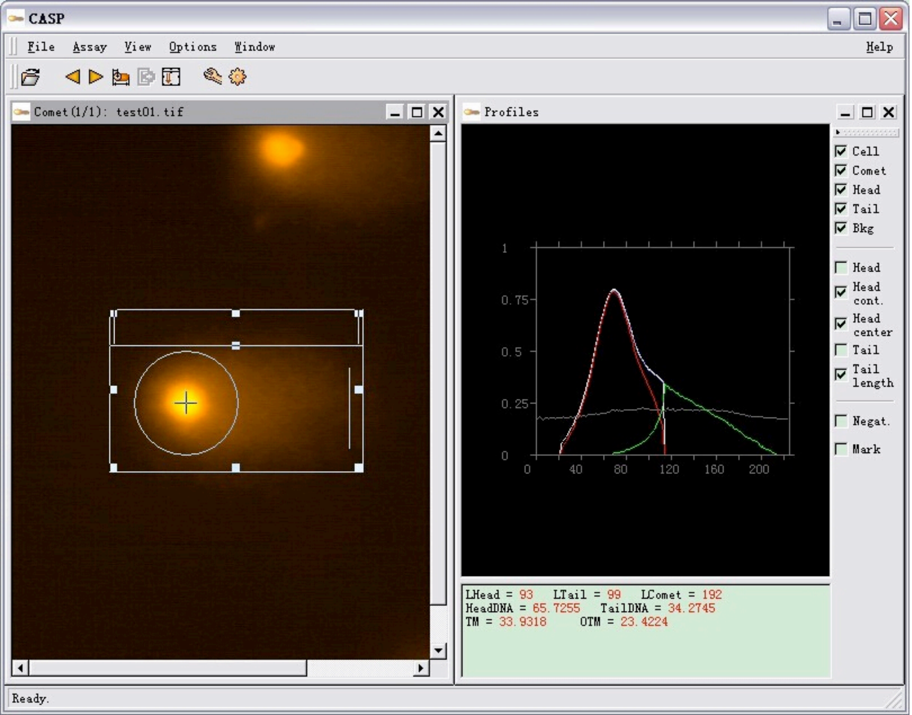
CASP
File Assay View Options Window Help
Comet (1/1): test01.tif
Profiles
Cell
Comet
Head
Tail
Bkg
Head
Head cont.
Head center
Tail
Tail length
Negat.
Mark
1
0.75
0.5
0.25
0
0 40 80 120 160 200
LHead = 93 LTail = 99 LComet = 192
HeadDNA = 65.7255 TailDNA = 34.2745
TM = 33.9318 OTM = 23.4224
Ready.
